# Immunohistochemical analysis in ethinylestradiol-treated breast cancers after prior long-term estrogen-deprivation therapy

**DOI:** 10.1186/s40064-015-0851-8

**Published:** 2015-03-05

**Authors:** Yoko Omoto, Takashi Takeshita, Yutaka Yamamoto, Mutsuko Yamamoto-Ibusuki, Mitsuhiro Hayashi, Aiko Sueta, Saori Fujiwara, Tetsuya Taguchi, Hirotaka Iwase

**Affiliations:** Department of Breast and Endocrine Surgery, Graduate School of Medical Sciences, Kumamoto University, 1-1-1 Honjo, Kumamoto, 860-8556 Japan; Department of Endocrinological and Breast Surgery, Graduate School of Medical Science, Kyoto Prefectural University of Medicine, 465 Kajii-cho, Hirokoji Agaru, Kawaramachi-dori, Kamigyo-ku, Kyoto, 602-0841 Japan; Department of Breast Surgery, Tanabe Central Hospital, 6-1-6, Tanabe-Chuo, Kyotanabe-city, Kyoto 610-0334 Japan

**Keywords:** Ethinylestradiol, Estrogen receptor, Metastatic breast cancer, Endocrine therapy, Estrogen treatment resistance

## Abstract

**Background:**

Estrogen receptor (ER) positive breast cancer can often be treated by hormone therapy; however a certain population of ER-positive patients become resistant to hormone therapy after long-term hormone treatment. Ethinylestradiol (EE2) is a derivative of estrogen, which has shown promising effects in these patients.

**Methods:**

We successfully obtained tissue samples from 6 patients undergoing EE2 treatment and examined 13 well-known breast cancer-related factors by immunohistochemistry. Of the 6 patients, 5 responded but one patient did not.

**Results:**

Before EE2 treatment, staining for both ER and androgen receptor (AR) was strong in the nucleus, and the progesterone receptor (PgR) was almost no staining. EE2 treatment significantly down-regulated ER and up-regulated PgR while nuclear and cytosolic AR were oppositely down- and up-regulated, respectively. Cytosolic staining of BRCA1 was significantly up-regulated by EE2 whereas nuclear staining tended to decrease. Individual comparisons suggested less induction of PgR and decreasing AKT but increasing pAKT in the non-responder following EE2 treatment.

**Conclusions:**

Our observations revealed that EE2 activated ER downstream genes; however it did not stimulate cell growth. This suggests that hormone resistant cells might receive growth signals from a non-genomic pathway and this may be reflected in their sensitivity to EE2 treatment.

**Electronic supplementary material:**

The online version of this article (doi:10.1186/s40064-015-0851-8) contains supplementary material, which is available to authorized users.

## Background

Estrogen receptor (ER) positive breast cancer is considered to be a low risk form of the disease and anti-estrogen treatment is generally applied even though this disease can be recurrent, if not life-threatening (NCCN guidelines® http://www.nccn.org/).

There are several different types of anti-estrogenic agents; Tamoxifen is an antagonist of ER, and has been widely used to treat cancer in both pre- and post-menopausal women. Aromatase inhibitors (AIs) are the representative anti-estrogenic agents in post-menopausal women, and are the first choice for patients after menopause. There are several different AI agents available; the non-steroidal AIs: anastrozole and letorozole, and the steroidal AI: exemestane. Fulvestrant is an estrogen receptor antagonist with no agonist effects, which works by down-regulating the estrogen receptor (Kansra et al. 2005). These agents generally inhibit ER function by either decreasing production of estrogen or by blocking the ER itself.

Ethinyl estradiol (EE2) is a derivative of 17β-estradiol (E2), which is the major endogenous estrogen in humans, and exhibits estrogenic activity. EE2 is used in many formulations of combined oral contraceptive pills (Hatcher RA 2004). In addition it has also been prescribed for patients with prostate cancer for many years, because administration of EE2 causes a reduction of androgen production (Dorner et al. 1985).

Very interestingly, EE2 administration has been shown to be effective against the recurrence of ER positive breast cancer after long-term anti-estrogen treatment (Iwase et al. 2013; Ellis et al. 2009). It was believed that estrogen stimulates the growth and development of ER-positive breast cancer, so this finding is quite the opposite to our consensus and the effects are something of a paradox.

To investigate this effect in more detail, we set up a clinical trial to evaluate EE2 treatment in our department. We registered 18 patients who were prescribed EE2 treatment after long-term AI treatment, and so far successfully obtained 23 tissue samples from 6 patients. Using immunohistochemistry, we analyzed the expression of known breast cancer-related genes in this study and examined the effects of EE2.

## Results

**Figure 1:**
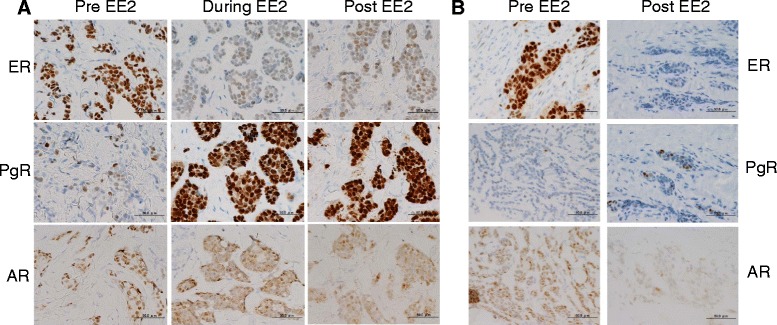
**Representative staining of ER, PgR and AR in patients pre, during and post EE2 treatment. A**: Staining in patient 4, who responded to EE2. Before EE2 treatment, ER staining was high, PgR staining was low and AR staining was only observed in the nucleus. After administration of EE2, ER decreased, PgR was increased and AR in the nucleus reduced while cytosolic staining increased. **B**: Staining in patient 3, who did not respond to EE2. Before EE2 treatment, high ER, low PgR and high nuclear AR were observed, while after EE2, ER was dramatically decreased as in patient 4. However, PgR was not induced and nuclear AR was decreased and not translocated to the cytosol.

ER, progesterone receptor (PgR) and Ki67 staining were observed only in the nucleus, while human EGFR-related 2 (Her2), Fas, transforming growth factor beta receptor 1 (TGFβR1) and insulin-like growth factor I receptor beta (IGF1Rb) were found on the cell membrane and phosphoinositide 3-kinase (PI3K) was localized in the cytosol. Androgen receptor (AR), breast cancer susceptibility gene I (BRCA1), protein kinase B (AKT) and phosphorylated AKT (pAKT) staining were observed in both the nucleus and the cytosol. Representative ER, PgR and AR staining are shown in Figure 1. Nuclear and cytosolic staining of each sample was evaluated by histo-score (HS) and cell membrane staining was categorized into 4 groups as described in Materials and methods. Results from all 18 samples are shown in Table 1.

**Table 1:** **Staining results of each samples**

		**ER**	**PgR**	**Ki67**	**BRCA1**		**AR**		**IGF1Rb**	**P13K**		**AKT**		**pAKT**		**TUNEL**	**HER2**	**Fas**	**TGFßR1**	**GF1Rb**
**Patient**		**nuc**	**nuc**	**nuc**	**nuc**	**cyto**	**nuc**	**cyto**	**cyto**	**nuc**	**cyto**	**nuc**	**cyto**	**nuc**	**cyto**	**m**	**m**	**m**	**m**	**m**
1	pre	158	8	10	121	0	94	0	80	0	46	18	95	5	90	25	1	0	0	3
	post	120	10	10	106	95	5	60	70	0	56	0	90	5	0	0	1	10	1	1
2	pre	156	0	20	97	0	124	50	80	0	106	10	42	0	0	0	1	0	2	1
	post	110	137	40	4	0	3	0	0	0	109	46	85	0	0	9	1	0	1	3
3	pre	140	6	20	55	0	111	0	90	0	100	105	0	0	40	6	1	0	1	3
	post	0	30	50	39	0	5	0	100	0	100	31	0	133	90	6	3	0	1	1
4	pre	156	17	10	116	0	106	0	0	0	88	40	90	0	0	0	1	0	0	1
	during	11	203	20	122	0	18	95	0	0	100	85	100	0	0	0	1	0	0	2
	post	10	122	30	100	100	15	98	0	0	100	95	95	0	0	3	0	0	1	2
5	pre	172	0	5	100	0	128	0	0	0	15	0	95	16	90	2	2	0	2	1
	pre	198	0	3	100	0	109	0	70	0	100	10	80	5	0	0	1	0	1	1
	pre	117	0	20	101	0	88	0	0	0	100	90	90	0	24	9	1	0	1	1
	pre	162	0	15	98	0	112	0	85	0	100	70	90	0	0	3	1	0	1	1
	pre	203	0	10	98	0	120	0	20	0	80	50	80	0	15	0	1		1	1
	during	89	141	5	45	0	101	100	90	0	95	19	60	0	0	0	2	0	1	1
	post	103	0	20	98	98	100	98	0	0	100	19	95	0	95	0	2	0	1	1
6	pre	205	161	10	80	0	200	90	100	0	98	50	90	46	0	6	1	0	1	3
	during	104	170	5	5	0	107	99	0	0	100	3	90	0	0	6	1	0	1	1

Nuclear and cytosolic staining were evaluated by HistoScore. Membrance staining was clasiffied as 0, 1+, 2+ and 3+ following the recommendations for Her2 staining.

nuc: nuclear, cyto: cytosol, m: membrane.

### Comparison of staining between groups at different time-points during EE2 treatment

Statistical comparisons were performed in two ways; one was to compare between Pre EE2 and (During+Post) EE2 treatment (Table 2A), the other comparison was between all 3 groups, Pre, During and Post EE2 treatment (Table 2B). In both comparisons, the expression of 4 genes appeared to be regulated by EE2 treatment. ER staining was quite strong before EE2 treatment; however it was significantly down-regulated by EE2 treatment. In contrast virtually no PgR staining was detectable before EE2 treatment, but it was significantly up-regulated after treatment. Before EE2 treatment, strong AR staining could be observed but was almost completely restricted to the nucleus; however nuclear AR staining was decreased and cytosolic AR was increased by EE2. BRCA1 staining was observed only in the nucleus before and during EE2 treatment, however EE2 significantly increased cytosolic staining of BRCA1 while nuclear staining tended to decrease. Staining of other proteins was not changed by EE2 treatment.

**Table 2:** **Statistical evaluation of nuclear and cytosol staining between different time points**

**A**		**Pre EE2 (n=10) mean (S.E.)**	**During + Post EE2 (n=8) mean (S.E.)**	**P-value**	**B**		**Pre EE2 (n=10) mean (S.E.)**	**During EE2 (n=3) mean (S.E.)**	**Post EE2 (N=5) mean (S.E.)**	**P-value**
ER	nuc	166.7 (9.0)	68.3 (18.2)	0.0006**	ER	nuc	166.7 (9.0)	68.0 (28.8)	68.6 (26.1)	0.0024**
PgR	nuc	19.2 (15.8)	113.1 (24.1)	0.01*	PgR	nuc	19.2 (15.8)	171.3 (17.9)	78.2 (26.8)	0.0113*
Ki67	nuc	12.3 (1.96)	22.5 (5.82)	0.2	Ki67	nuc	12.3 (1.96)	10.0 (5.0)	30.0 (7.07)	0.052
BRCA1	nuc	96.6 (5.79)	64.8 (16.7)	0.3	BRCA1	nuc	96.6 (5.79)	57.3 (34.3)	69.4 (20.3)	0.56
	cyto	0 (0)	36.6 (17.8)	0,047*		cyto	0 (0)	0 (0)	58.6 (23.9)	0.0126*
AR	nuc	119.2 (9.79)	44.2 (17.2)	0.039*	AR	nuc	119.2 (.979)	75.3 (28.7)	25.6 (18.7)	0.0065**
	cyto	14.0 (9.79)	68.75 (15.7)	0.0097**		cyto	14.0 (9.79)	98.0 (1.52)	51.2 (22.0)	0.0092**
PI3K	cyto	83.3 (9.39)	95.0 (5.73)	0.31	PI3K	cyto	83.3 (9.3)	98.3 (1.66)	93.0 (9.41)	0.53
AKT	nuc	44.3 (11.3)	37.2 (12.6)	0.72	AKT	nuc	44.3 (11.3)	35.6 (25.0)	38.2 (16.0)	0.9
	cyto	75.2 (9.69)	76.8 (11.8)	0.58		cyto	75.2 (9.69)	83.3 (12.0)	73.0 (18.3)	0.81
pAKT	nuc	7.2 (4.5)	17.2 (16.5)	0.63	pAKT	nuc	7.2 (4.5)	0 (0)	27.6 (26.3)	0.44
	cyto	25.9 (11.4)	23.1 (15.1)	0.57		cyto	25.9 (11.4)	0 (0)	37.0 (22.6)	0.36
IGT1Rb	cyto	52.5 (13.3)	32.5 (16.1)	0.37	IGT1Rb	cyto	52.5 (13.3)	30.0 (30.0)	34.0 (21.4)	0.64
TUNEL		5.1 (2.42)	3.0 (1.26)	0.74	TUNEL		5.1 (2.44)	2.0 (2.0)	3.6 (1.74)	0.75

The Wilcoxon test/the Kruskal-Wallis test was used to analyze the significance of differences between groups.

nuc: nuclear, cyto: cytosol.

*<0.05, **<0.01.

When expression of membrane proteins; Her2, Fas, TGFβR1 and IGF1Rb were analyzed, no differences were observed between groups (Data not shown).

### Comparison of staining between individuals at different time-points during EE2 treatment

**Figure 2:**
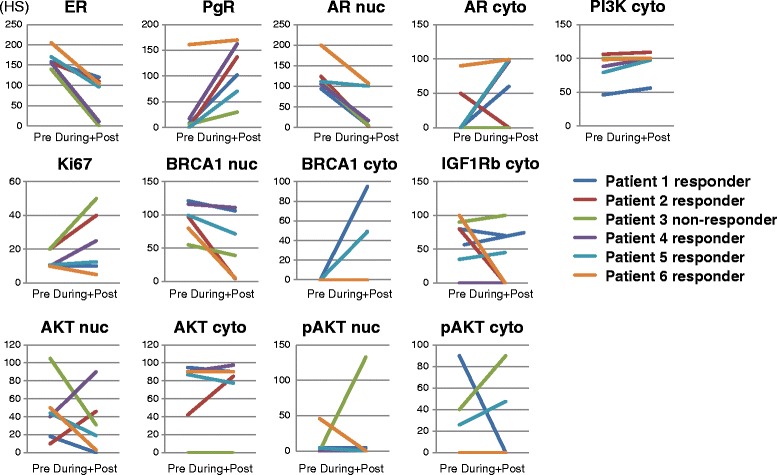
**Comparison of expression between Pre and (During+Post) EE2 treatment in individual patients.** Patients 1,2,4,5 and 6 responded to EE2 treatment whereas patient 3 did not. The average HS was calculated for a patient if they had several samples in a group.

The therapeutic effect and duration of EE2 treatment were: patient 1: responder 14 m, patient 2: responder 14 m, patient 3: non-responder 4 m, patient 4: responder 12 m, patient 5: responder 8 m, and patient 6: responder still receiving ongoing treatment for more than 14 m. To investigate differences in the regulation of selected genes underlying the different therapeutic effects, we compared differences in expression between Pre- and (During+Post) treatment with the exception of membrane staining. Results are shown in Figure 2. This study was not evaluated statistically because there was only one patient who did not respond to EE2, however, this patient appeared to show a different pattern of PgR expression to the responders, with no increase after EE2 treatment even though ER expression was drastically reduced, AKT expression seemed to decrease whereas pAKT expression increased after treatment both in the nucleus and the cytosol but especially in the nucleus.

## Discussion

Hormone therapy is important in breast cancer treatment and around 70% of patients are categorized as suitable for hormone therapy (American Cancer Society 2013http://www.cancer.org/cancer/breastcancer/detailedguide/breast-cancer-treating-hormone-therapy). Hormone treatment can be administered orally or by one shot injection, which has less influence on bodily functions so that patients can continue daily life as they are used to (Iwase 2008). There are a variety of different medicines currently available, including anastrozole, letrozole and exemestane as AIs; tamoxifen and raloxifene as estrogen receptor modulators; and fulvestrant as an ER downregulator. Consequently, even if a patient fails to respond to one type of therapy, they can be switched to another medicine with a different structure or method of action and if that also fails, they can again be switched to another medication. Hormone therapy allows patients to maintain a better quality of life for a certain duration before chemotherapy is applied (Carlson et al. 2012).

In breast cancer samples, ER and PgR expression status are evaluated by immunohistochemistry. If a sample shows more than 1% staining for one of these receptors, it is classified as hormone receptor-positive and hormone treatment is applicable (Hammond et al. 2010). Comprehensive gene analysis has allowed breast cancers to be categorized according to their intrinsic subtype and Luminal A, a group with high expression of the ER, is considered to be highly responsive to hormone therapy (Sorlie et al. 2001). Therefore, ER and PgR status are very important for the application of hormone therapy, especially ER status. However, approximately 30% of ER-positive cases do not respond favorably to hormone therapy (Rubens and Hayward 1980) and in addition a subset of the population may become resistant after long-term hormone treatment even though they remain ER positive (Jordan and Ford 2011).

Recently, it was reported that EE2 administration was effective in treating hormone therapy-resistant ER-positive breast cancer patients (Ellis et al. 2009; Iwase et al. 2013). EE2 is a derivative of estrogen, therefore this treatment sounds in opposition to endocrine therapy, which is considered to act by blocking estrogen function. This paradoxical therapy can postpone the necessity for administration of chemotherapy because after EE2 treatment, normal anti-estrogenic agents become effective again even if treatment with the same medication failed before.

The very best point to start EE2 treatment is not yet understood. In a trial of EE2 by Ellis et al., eligible patients were those with metastatic breast cancer treated with AIs at least 24 weeks progression-free survival, or relapse after two or more years of adjuvant AIs (Ellis et al. 2009). In our study, our inclusion criteria were: postmenopausal women with breast cancer (not less than 50 years old) who had progressive disease, and who were receiving sequential treatments with AIs or Selective estrogen receptor modulators (SERMs) (the university hospital medical information network (UMIN) Clinical Trials Registry (UMIN000002831) http://www.umin.ac.jp/ctr/index.htm). It would be a great benefit for ER-positive patients if we could distinguish between hormone therapy insensitivity and EE2 sensitivity. So far 18 patients have been registered for EE2 treatment in our department after long-term Als treatment, and have shown very interesting results; the response rate was 50% and the clinical benefit was 56% (Iwase et al. 2013). Furthermore, we successfully obtained 23 tissue samples from 6 patients. Therefore, first of all, we focused on 13 genes and factors, which are related to cancer progression and performed immunostaining to see how these genes were regulated during EE2 treatment in this study.

The genes which we selected could be divided into 4 categories, hormone-related factors: ER, PgR and AR; growth/proliferative factors: Her2, IGF1Rb, TGFβR1 and Ki67; intracellular signaling factors: AKT, pAKT and PI3K; and apoptosis-related factors: BRCA1, Fas and TUNEL.

Before EE2, i.e. under estrogen-depleted conditions, ER and AR staining was quite strong while virtually no PgR staining was observed, as we had expected. In a comparison among samples of pre-, during and post-treatment or between pre- and (during+post)-treatment, only ER, PgR, AR and BRCA1 showed any significant difference between the treatment groups. Comparison among pre-, during- and post-treatment or between pre- and (during+post)-treatment showed that ER was significantly down-regulated by EE2 treatment whereas PgR was up-regulated (Table 2A,B). Nuclear AR staining was down-regulated whereas cytosolic AR was up-regulated by EE2. ER, PgR and AR are members of the nuclear receptor superfamily and are closely related to estrogen. PgR is well-known as a down-stream gene of ER (Lin et al. 2004) therefore this result strongly suggests that EE2 activates ER signaling. AR has a strong relationship with ER either directly (Panet-Raymond et al. 2000; Peters et al. 2009) or by regulation of their ligands. Transportation and translocation of the AR to the nucleus is performed by its ligand androgen (Callaway et al. 1982). Decreasing androgen concentrations during EE2 treatment could be a reason for this observation; androgen levels were rather high due to AI treatment but decreased to relatively low levels after EE2 treatment as has been observed in prostate cancer treatment (Brunton 2006. The other notable finding was that cytosolic staining of BRCA1 was significantly up-regulated by EE2 whereas nuclear staining tended to decrease (Table 2). BRCA1 and BRCA2 are tumor suppressor genes and these mutations were strongly associated with hereditary breast cancer (Yoshida and Miki 2004; Duncan et al. 1998). BRCA1 is responsible for repairing DNA and when normally expressed it helps repair damaged DNA or destroys cells if the DNA cannot be repaired. BRCA1 staining was mainly observed in nuclei but was also present in the cytosol. BRCA1 nuclear staining was frequently reduced in breast tumor tissue compared to normal tissue. The protein stained in the cytosol was considered to be a mutated form of BRCA1 (Kashima et al. 2000; Tulchin et al. 2013) and loss of nuclear BRCA1 expression is associated with a highly proliferative tumor phenotype (Jarvis et al. 1998). Estrogen treatment upregulates BRCA1 expression but has no effect on cellular localization (Romagnolo et al. 1998). Our results showed that when EE2 treatment ends i.e. in the post-EE2 group, tumors became resistant to EE2 and started to grow again as shown by the Ki67 index which tended to increase after treatment. We were unable to identify the precise reason for BRCA1 translocation; however it may be related to tumor re-growth.

One interesting finding was that EE2 activated ER signaling, as we observed in PgR regulation; however, EE2 did not stimulate cell growth. Of the 6 patients in this study, 5 patients responded indicating that EE2 suppressed tumor growth. ER signaling occurs not only via the classical ER genomic pathway through the estrogen responsive element (ERE), but also by cross-talk between growth factor receptors, the so called non-genomic pathway, and might involve acquisition of hormone therapy resistance (Johnston 2010). Comparison between patients suggested the possibility of differences in the regulation of PgR and AKT/pAKT in the non-responsive patient (Figure 2). It has been reported that long-term estrogen depletion involves a possible contribution of the Akt pathway to the phosphorylation of ER (Fujiki et al. 2014) in breast cancer cells. We could speculate; 1^st^ that EE2 might act in the same manner as E2; however, transcriptional circumstances are changed during long-term hormone treatment; 2^nd^ that EE2 could show differences in conformation and induce recruitment of different co-factors as other SERMs do. Transcriptional pathways include not only the direct genomic pathway but also membrane or non-genomic pathways. Stimulation of another pathway or an imbalance of activation could affect the response to EE2 and hormone resistance. The results of gene expression profiling show wide variations between patients and we need more cases and samples to conclude whether long term estrogen depletion causes activation or inactivation of estrogen receptor signaling (Martin et al. 2005; Martin et al. 2003), Non-responder might have dysfunction of estrogen-ER signaling and might be stimulated cell proliferation by non-genomic pathway.

The biology of estrogen-depleted cells was investigated by cell culture, which indicated that estrogen depleted estrogen dependent cells increased ER expression and became sensitive to estrogen. These cells were able to grow at lower doses of estrogen (Martin et al. 2005). Additionally, treatment with estrogen within the normal range did not increase proliferation but induced apoptosis in estrogen-depleted cells (Martin et al. 2005). Recently it was reported that genomic mutation of the ER itself could occur during long-term hormone therapy (Jeselsohn et al. 2014). This mutated ER then becomes hypersensitive to estrogen and is able to grow even with super low amounts of estrogen, suggesting that this is one of the possible mechanisms by which these cells develop resistance to hormone therapy. Ellis et al. investigated the effects of different concentrations of EE2, 6 mg or 30 mg per day (Ellis et al. 2009) and reported that the effect of 6 mg was similar to that obtained with a dose of 30 mg. Therefore, the lower dosage of 6 mg was sufficient to examine its function further. In our study, all 18 patients were administered 6 mg per day and showed a response rate of 50% and a clinical benefit of 56% (Iwase et al. 2013).

## Conclusions

EE2 is a truly effective and beneficial agent; however we need to accumulate further knowledge to understand how to apply this medicine: its dosage, application and criteria. Additionally, the mechanism underlying the effects of EE2 acting through the ER is still not clearly understood.

In this study, the total number of samples was too small to analyze and reach a valid conclusion concerning the mechanism of hormone therapy resistance. We will continue to enter the data from the other patients in the EE2 trial. In the future, it will be obligatory to perform large scale gene expression profiling because an unknown gene or gene family may be involved in changing critical characteristics. Further investigation is awaited.

## Materials and methods

### Patients and samples

This EE2 trial was approved by the institutional review board of Kumamoto University Hospital and registered to UMIN center (UMIN000002831). This study was informed to all patients and we obtained consent to participate in the study and consent to publish. The criteria for registration have previously been described in detail (Iwase et al. 2013). Briefly, all patients had received prior sequential hormone therapies including chemotherapy. Each patient’s pretreatment history is shown in Table 3.

**Table 3:** **Characteristics of patients and the timing of tissue collection**

**Patient number/age**	**Previous treatment**																		**EE2 response/biopsy site**
		LHRHa	LHRHa		LHRHa														
1		+ Tam	+ ANA		+ L		TS-1	VNR		PTX		E		**EE2**		L	**EE2**		
		10m	12m		3m		12m	21m		3m		4m		**14m**		5m			Responder liver, local
59													**^**		**^**				
													**B**		**A**				
2			TS-1 +																Responder lymph node
		DTX	ANA		E		EC	DTX		XC		L		**EE2**		L	**EE2**		
		15m	13m		5m		13m	24m		6m		7m		**14m**		6m			
62													**^**		**^**				
													**B**		**A**				
3																	H +		Non-responder local
							FEC	XT		ANA		E		**EE2**		FUL	PTX		
							3m	12m		8m		2m		**4m**		3m			
56						**^**			**^**		**^**				**^**			**^**	
						B			B		**B**				**A**			A	
		E	MPA		Tam		L	TS-1		ANA		Tor		**EE2**		L			Responder skin
4			8m		9m		2m	13m		8m		2m		**12m**					
64									**^**		**^**			**^**	**^**				
									B		**B**			**D**	**A**				
		AC	H		L		Tor	E+H		PTX		L		**EE2**		FuL			Responder local
5		8m	7m		8m		6m	1m		4m		3m		**8m**					
58				**^**	**^**	**^**	**^**	**^**					**^**	**^**	**^**				
				B	**B**	**B**	**B**	**B**					**B**	**D**	**A**				
	L	ANA	XC		TS-1		ANA	Tor		PTX		ANA		**EE2**					Responder local
6	16m	3m	9m		3m		2m	7m		11m		3m		**14m** **--**					
83													**^**		**^**				
													**B**		**B**				

^: the time point when tissue was obtained.

B: before EE2 treatment D: During EE2 treatment; A: After EE2 treatment.

Sample shown in bold letter were used in this study.

*Abbreviations:* ANA: anastozole; AC: doxorubicine + cyclophosphamide; DTX: docetaxel; E: exemestane; EC: Epirubicine + cydophoshamide; FEC: cydoshsphamide + epirubine+5-FU; FUL: fulvestrant; H: herceptine; L: letrozole; MPA: medroxyprogesterone; PTX: paclitaxel; Tam: tamoxifen; Tor: toremifeme; VNR: vinorelubin; XC: capecitabine + cyclophosphamide; XT: capecitabine + docetaxel.

A total of 23 tissue samples were obtained from 6 patients; however, 4 pre-treatment samples and one post-treatment sample were not evaluated in this study to avoid the complicating effects of chemotherapy. Therefore, 18 tissues from 6 patients were used in this study. All patients had been administered EE2 after long-term treatment with multiple anti-hormone agents. Tissue samples were collected by core needle biopsy from metastatic lesions of patients who had undergone EE2 treatment at certain clinical points, as shown in Table 3. Of the 18 samples, 10 samples were obtained before EE2 treatment, 3 were collected during treatment and 5 were obtained after treatment. All samples were used for the immunohistochemical (IHC) study to compare expression during this time period.

### Antibody, immunohistochemical methods and evaluations

A total of 13 different staining procedures were performed in this study, including immunostaining for 11 breast cancer-related genes plus one antibody to detect phosphorylated protein and TdT-mediated dUTP nick end labeling (TUNEL). These 13 targeted proteins or markers are functionally categorized into 6 groups: nuclear receptors (ERα, PgR, AR); growth factors (Her2, IGF1Rb, TGFβR1); tumor suppressor genes (BRCA1); cell proliferation (Ki-67, TUNEL); apoptosis related (Fas); intracellular signal transduction (AKT, pAKT, PI3K). Information on all the antibodies is shown in Table 3.

All formalin-fixed, paraffin-embedded specimens were cut into 4-μm sections, deparaffinized, heated 3 times for 5 min each in citrate buffer (pH 7) in a 1,000 W microwave for antigen retrieval and incubated for 10 min in distilled water containing 3% hydrogen peroxide. The primary antibody was applied after blocking, and incubated at 4°C overnight. Detection and visualization was performed by several methods as indicated in Table 4, according to the manufactures’ protocol. As a negative control, parallel sections were immunostained without exposure to primary antibodies. No immunoreactivity was observed in these sections.

**Table 4:** **List of antibodies and methods of visualization**

	**Antibody**	**Source**	**Dilution**	**Visualization**
ER	1D5 (Dako)	Mouse monoclonal	1:50	Histofine Simple stain MAX-PO° (Nichirei)
PgR	PgR636 (Dako)	Mouse monoclonal	1:800	Histofine Simple stain MAX-PO° (Nichirei)
AR	NCL-AR-318 (Leica)	Mouse monoclonal	1:100	VECTASTAIN Elite ABC (Vector)
Ki67	MIB-1 (Dako)	Mouse monoclonal	1:50	I-VIEW DAB universal kit (Roche)
BRCA1	MS110 (Abcam)	Mouse monoclonal	1:200	Histofine Simple stain MAX-PO° (Nichirei)
IGF1Rb	#3027 (CST)	Rabbit polyclonal	1:600	VECTASTAIN Elite ABC (Vector)
TGFβR1	8A11 (Abcam)	Mouse monoclonal	1:100	VECTASTAIN Elite ABC (Vector)
Fas	C18C12 #4233 (CST)	Rabbit monoclonal	1:480	SignalStain° boost (CST)
AKT	11E7 #4685 (CST)	Rabbit monoclonal	1:100	SignalStain° boost (CST)
pAKT (Ser473)	D9E #4060 (CST)	Rabbit monoclonal	1:50	SignalStain° boost (CST)
PI3K p85	M253 (Abcam)	mouse monoclonal	1:200	VECTASTAIN Elite ABC (Vector)
Her2	anti-HER2 (4B5) (Roche)	Rabbit monoclonal		I-VIEW DAB universal kit (Roche)
TUNEL	TUNEL Apoptosis Detection Kit (Millipore)			

This study was reported according to the Reporting Recommendations for Tumor Marker Prognostic Studies (REMARK) criteria (McShane et al. 2005). Nuclear staining and cytoplasmic staining were independently scored by HS. The HS represented the product of the staining intensity (0: negative, 1: weak, 2: moderate and 3: strong) and the percentage of positive cells (0–100%) for each sample, with a maximum HS of 300. We counted approximately 100 cancer cells in five randomly-chosen microscopic fields. Cytomembrane staining was evaluated according to the recommendation for Her2 staining (Wolff et al. 2013), with staining categorized into scores of 0, 1+, 2+ and 3+.

### Statistical analysis

The nuclear or cytosolic staining of each protein was statistically analyzed. The Wilcoxon signed-rank test was used for comparisons between 2 groups and the Kruskal-Wallis test was used for 3 groups. Membrane staining was analyzed using Student’s t-test between groups. *P* values < 0.05 were considered a significant result. All analyses were performed using JMP software version 10.0.1 for Windows (SAS institute Japan, Tokyo, Japan).

## Abbreviations

AI: Aromatase inhibitor
AKT: Protein kinase B
AR: Androgen receptor
BRCA1: Breast cancer susceptibility gene I
E2: 17β-estradiol
EE2: Ethinyl estradiol
ER: Estrogen receptor
ERE: Estrogen responsive element
Her2: Human EGFR-related 2
HS: Histo-score
IGF1Rb: Insulin-like growth factor I receptor beta
pAKT: Phosphorylated AKT
PgR: Progesterone receptor
PI3K: Phosphoinositide 3-kinase
REMARK: Recommendations for Tumor Marker Prognostic Studies
SERMs: Selective estrogen receptor modulators
TGFβR1: Transforming growth factor beta receptor 1
TUNEL: TdT-mediated dUTP nick end labeling
UMIN: The University Hospital Medical Information Network
